# Laser-Induced Generation of Hydrogen in Water by Using Graphene Target

**DOI:** 10.3390/molecules27030718

**Published:** 2022-01-22

**Authors:** Wieslaw Strek, Przemysław Wiewiórski, Włodzimierz Miśta, Robert Tomala, Mariusz Stefanski

**Affiliations:** Institute of Low Temperature and Structure Research, Polish Academy of Sciences, 50-422 Wroclaw, Poland; w.strek@intibs.pl (W.S.); pwiewiorski@gmail.com (P.W.); w.mista@intibs.pl (W.M.); r.tomala@intibs.pl (R.T.)

**Keywords:** hydrogen generation, graphene foam, white light emission, laser irradiation

## Abstract

A new method of hydrogen generation from water, by irradiation with CW infrared laser diode of graphene scaffold immersed in solution, is reported. Hydrogen production was extremely efficient upon admixing NaCl into water. The efficiency of hydrogen production increased exponentially with laser power. It was shown that hydrogen production was highly efficient when the intense white light emission induced by laser irradiation of graphene foam was occurring. The mechanism of laser-induced dissociation of water is discussed. It was found that hydrogen production was extremely high, at about 80%, and assisted by a small emission of O_2_, CO and CO_2_ gases.

## 1. Introduction

Recently, hydrogen production has become particularly important due to the need to produce green energy and, thus, reduce CO_2_ emissions resulting from the combustion of fossil fuels to the atmosphere. Therefore, the scientific community worldwide is working intensively to obtain hydrogen from different sources, such as: biomass [1,2,3], oil [4,5,6] or methane [7,8,9] reforming, biological sources [10,11,12] and coal gasification [13,14,15]. It is also worth mentioning that electrolysis of water is the most efficient method for large-scale hydrogen production applied in carbon-free energetics [16,17,18,19,20]. Taking into account the type of electrolyte and operating conditions, four types of electrolysis can be distinguished: polymer electrolyte membrane (PEM) water electrolysis [21,22], solid oxide electrolysis [23,24], microbial electrolysis cells [25,26] and alkaline water electrolysis [27,28]. The production of hydrogen from water and hydrocarbon liquids using a pulsed laser was a subject of several publications [29,30,31,32,33,34,35].

The splitting of water by electrolysis is related to the formula:

2H_2_O → 2H_2_ + O_2_ (1)

A generation of hydrogen from water and carbon due to intense laser irradiation was discussed by Akimoto et al. [30] in terms of the steaming process of coal at high temperatures (~800 °C). The interaction of water with carbon gives access to the synthesis of carbon oxide, carbon dioxide and oxygen, according to the following steps:

C + H_2_O → H_2_ + CO (2)

C + 2H_2_O → 2H_2_ + CO_2_ (3)

C + 3H_2_O → 3H_2_ + CO + O_2_ (4)

In our earlier experiments with methanol [36] and ethanol [37], we observed that the efficient generation of hydrogen occurred when graphene demonstrated intense white light emission under laser irradiation. This emission resulted from the multiphoton ionization of graphene. The multiphoton ionization is assisted by the emission of hot electrons and precedes the broadband luminescence combined with the (sp^2^,sp^3^)→(sp^3^,sp^2^) hybrid transition [38,39].

In the presence of graphene, the process of laser-induced splitting of water may be schematically depicted:

(5) $$\langle C\rangle +N\;h\nu \;\to \;{\langle C\rangle }^{+}+e^{-}$$

2e^−^ + 2H_2_O → 2H_2_ + O_2_ (6)

where N is the number of photons hν necessary for multiphoton ionization of graphene, 〈C〉 and 〈C〉^+^ denote graphene and a single ionized graphene molecule, respectively.

Distilled water is a poor electrical conductor, and after admixing NaCl, it becomes an electrolyte solution (saline) conducting electricity [40]. It is known that hydrogen production by the hydrolysis method is much more efficient for saline compared to distilled water [16,41].

In the present work, we report experiments of laser-induced generation of hydrogen from water and saline by using graphene aerogel as a photocatalyst and CW infrared laser diode for excitation. The generation of H_2_ was a dominant process and increased with excitation laser power, contrary to the generation of O_2_, CO and CO_2_ gases. The emission of the last two molecules was due to graphene degradation. In this paper, the mechanism of laser-induced hydrogen production in water is discussed in terms of the multiphoton ionization of graphene, accompanied by intensive emission of white light and ejection of hot electrons responsible for the dissociation of water molecules.

## 2. Results and Discussion

The experiments on hydrogen generation from water were performed using laser irradiation of immersed graphene aerogel (GA) treated as a target. It was found that graphene under irradiation with a focused laser beam demonstrated an intense white light emission. The process is a point emission, occurring only in a spot of focused laser beam [38,39]. The emission is due to the multiphoton absorption, leading to ionization of carbon atom C, and it may be schematically described by the following formula:

(7) $$C+N\;\hbar \omega \;\to \;C^{+}+e^{-}+\mathrm{WE}$$

where C^+^ is a single ionized carbon, e^−^ is an ejected electron, N determines the number of photons necessary for the multiphoton ionization, and WE denotes observed white light emission. Light emission is preceded by the ejection of hot electrons, giving access to the dissociation of water molecules on the surface of graphene aerogel, because the dissociation energy of the water molecule (9.8 eV) is higher than the work function of graphene (4.5 eV). 

**Figure 1:**
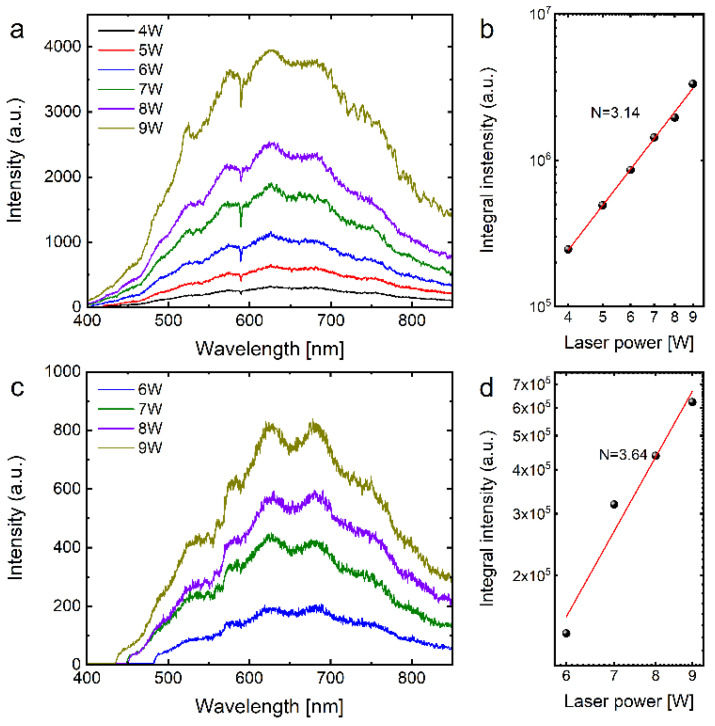
The emission spectra of laser-irradiated graphene foam with different excitation laser power in saline (**a**,**b**) and distilled water (**c**,**d**). The narrow dips observed at ~589 nm in the emission spectrum of saline water may be assigned to the Na^+^ ions due to the dissociation of NaCl. They were not seen for distilled water.

In the course of the experiments, the laser-induced white emission (LIWE) process was not stable for graphene aerogel immersed in distilled water and irradiated by CW LD, even at low excitation power. It was found that the total intensity of white emission was lower by more than one order of magnitude compared to saline. The respective plots of excitation power dependences of the LIWE intensities for GA in distilled water and saline are shown in Figure 1.

Graphene is a hydrophobic material, and the interaction of the laser beam with its surface significantly limits the ejection of electrons from the GA surface and reduces the emission of photons. NaCl salt significantly stabilizes and increases light emission from the GA surface because saline becomes electrolyte, and hot electrons increase the dissociation of water molecules. The LIWE process is very stable at 1% of saline water and more intense by at least one order of magnitude.

It should be noted that the electric characteristics of NaCl solution in distilled water strictly depend on the dynamics of the phenomena occurring in them [42]. Although CW laser irradiation was used from a DC source, the dynamic characteristics of water should be taken into account, which changes its properties significantly with increasing electrical activity near LIWE spot on graphene. The gas products of laser-induced decomposition of distilled water and saline measured in an argon atmosphere as a function of excitation laser power are listed in Table 1 and Table 2, respectively.

One can see that with increasing excitation laser power for distilled water, the amount of H_2_ significantly decreases for the excitation power density greater than 9 W. According to Table 1, H_2_ reached 47.00% for excitation power 10 W, while the second fraction—CO—was equal to 31.33%. The other gases, namely O_2_ and CO_2_, were emitted in much smaller amounts, i.e., ~11%. 

Table 2 shows that the percentage share of hydrogen in the saline increases from 57.14% to 80.91% for an excitation power increasing from 4.5 W to 5.5 W. Quite the opposite trend was observed for O_2_, CO and CO_2_ gases, gradually decreasing with laser power. It is important to note that the content of CO_2_ was extremely small (4.84%) for excitation power 5.5W. An increase in laser power significantly increases the multiphoton ionization of graphene assisted by intense white emission and ejection of hot electrons. An increase in white emission and assisted electron emission is exponentially dependent on excitation laser power. Therefore, a high generation of electrons is responsible for a strong increase in hydrogen generation.

It can be observed that with increasing laser power in distilled water, the formation of CO increases, while the amount of H_2_ decreases. This phenomenon probably occurs because with increasing laser power, an increase in O_2_ generation is observed, leading to higher binding of CO in relation to CO_2_. It is associated with greater binding energy of CO than of CO_2_. One can note that, with increasing laser power, an amount of O_2_ increases in contrast to H_2_ that decreases. The opposite situation is observed in the presence of NaCl. One can suppose that in saline, the ejection of electrons is higher compared to distilled water because ionization manifested in white light emission and emission of electrons is almost five times more efficient.

**Figure 2:**
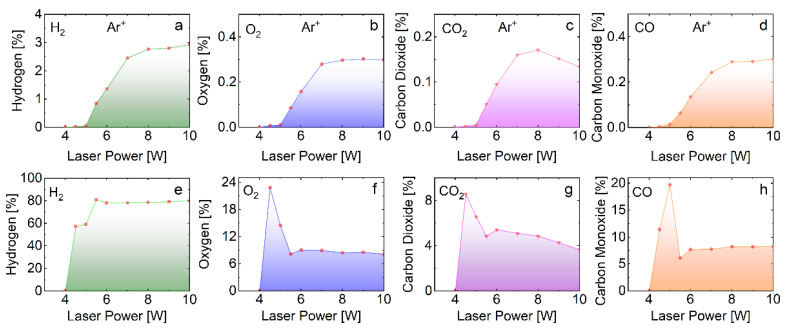
The power dependence of gas products in (**a**–**d**) and without (**e**–**h**) the presence of Ar, resulting from laser irradiation of H_2_O +1% NaCl.

The power dependence of laser-induced decomposition of H_2_O +1% NaCl into H_2_, O_2_, CO and CO_2_ gases, is shown in Figure 2.

**Figure 3:**
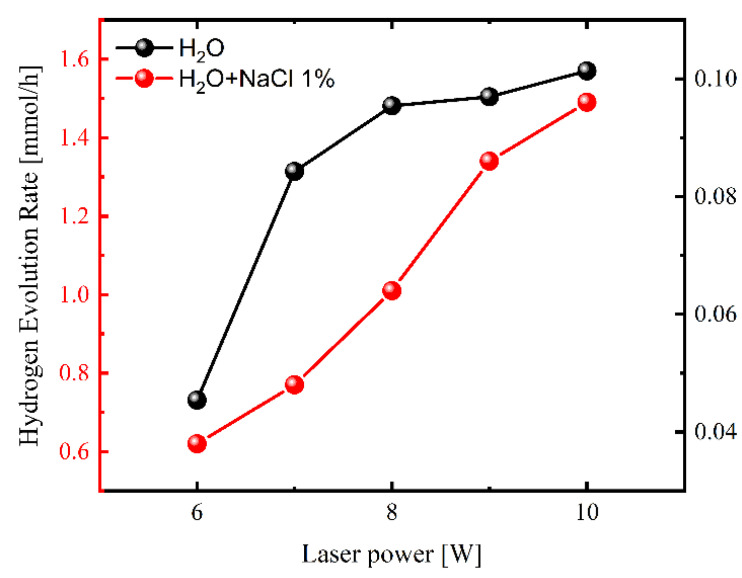
Hydrogen evolution rate from saline and distilled water by laser irradiation of graphene aerogel.

The excitation laser power dependence of the hydrogen evolution rate of distilled and saline water is shown in Figure 3. The total flow of H_2_ was determined to be 1.61 mmol/h and 0.10 mmol/h under the highest power of applied IR laser 10W for saline and distilled water, respectively.

**Figure 4:**
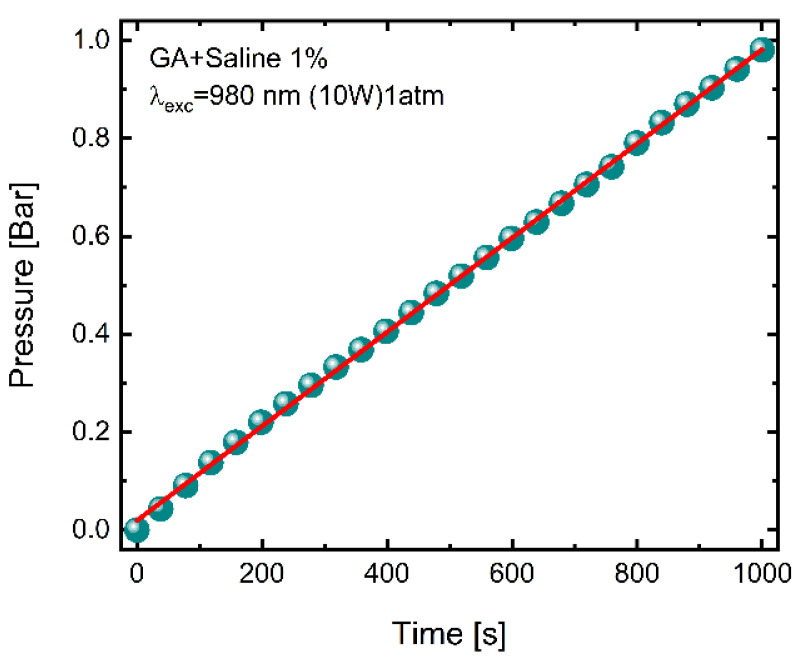
The increase in total gas pressure during water splitting in the closed cuvette after long-time exposure.

Figure 4 shows the increase in total pressure in the closed cuvette during the first cycle of the water-splitting process. The rise in pressure by 1 bar was obtained after about 1000 s. The higher gas pressures over 1 bar in the cuvette were not measured. The relative saline-splitting compression ratio (α)—estimated at about 1 mBar/sec—is relatively high.

The effective laser-induced hydrogen generation from water with carbon powder by using Nd:YAG pulse laser irradiation in Vis and NIR was reported by Akimoto and Maeda [30]. The authors obtained hydrogen generation at a level of 33% in the air atmosphere and carbon monoxide at 11%. In argon, the amount of hydrogen was higher, about 48.7%, and CO was 20.5%. The content of CO_2_ was almost negligible, below 1%. In our experiment, the amount of hydrogen was much higher, reaching nearly 81% and with small amounts of oxygen O_2_ ~8%, carbon monoxide CO ~5% and carbon dioxide CO_2_ ~6% for excitation power 5.5 W. It is important to stress that the efficient laser-induced generation of hydrogen by using CW infrared laser diode occurred due to efficient emission of white light assisted by the ejection of hot electrons, initiating the generation of hydrogen.

## 3. Materials and Methods

**Figure 5:**
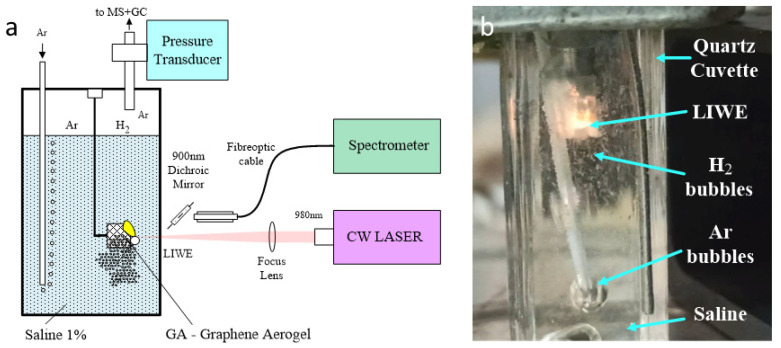
Experimental set-up for hydrogen generation from water, using graphene as a photocatalyst (**a**); Photo of the cuvette of water with immersed graphene scaffold irradiated with 980 nm laser beam (**b**).

The experiments were performed in a quartz cuvette filled with water. The 3D graphene foam scaffold (synthesized according to the procedure described in details in [39]) immersed in water was subjected to irradiation with a focused laser beam (see Figure 5). The CW laser diode (LD) 980 nm was used as an excitation source for the experiment. The 3D graphene foam scaffold applied in the study is shown in Figure 5b. Gas analysis was performed using mass spectrometer Pfeiffer Vacuum OmniStar QMS 200 (Asslar, Germany). Gas flow was managed by Brooks Instrument 5860E series mass flow controllers (Hatfield, PA, USA). Additional calibrations were performed using GC-MS Perkin Elmer Clarus 680 SQ8S (Waltham, MA, USA) and Agilent GC HP 6890 (Santa Clara, CA, USA). 

It is worth mentioning that there are a two variants of hydrogen generation: “inflow mode”—using Ar as a carrier gas—and “batch process” in a closed overpressure system. At atmospheric pressure, the laser exposure time was 60 s—constant for all tests. The next variant was a closed pressurized system (batch process) predisposed for assumed total of 1 bar of relative pressure (i.e., 2 bars with atmospheric pressure). The relative error of gas concentration was 5% due to repeatability of the following laser irradiations.

The conductivity and pH parameters of saline and distilled water used in the experiment were measured by us to be 1.60 × 10^4^ µS/cm (pH = 5.8) and 10 µS/cm (pH = 7), respectively. It is important to note the conductivity of saline water increased by almost three orders of magnitude relative to distilled water. The experiments on laser-induced hydrogen generation were performed for distilled water and 1%wt NaCl dissolved in H_2_O (saline).

## 4. Conclusions

In this paper, we have reported the laser-induced hydrogen generation from water by using graphene foam as a photocatalyst. The experiments were performed for distilled H_2_O and saline. The results showed that hydrogen production was much more efficient for salted water. As a result of laser irradiation, the main gas products were hydrogen, oxygen, carbon oxide and carbon dioxide. It was found that the process was characterized by threshold behavior and was strongly dependent on excitation laser power. Hydrogen generation increased strongly with increasing laser power in a range of 4.5–5.5W. The fraction of hydrogen significantly increased compared to other gases, such as oxygen, carbon oxide and carbon dioxide. The percentage of generated hydrogen for salted water reached nearly 81% compared to distilled water at 47%. It is the highest-efficiency process of laser-induced hydrogen generation from water reported in the literature. The technology of laser-induced hydrogen generation by using the relatively stable and cheap high-power laser diodes and graphene as a photocatalyst seems to be very promising for applications in the construction of small-scale hydrogen generators, coupled directly to fuel cells.

## Figures and Tables

**Table 1:** The ratio of gas products of laser-induced decomposition of distilled water in a function of applied laser power in an argon atmosphere.

Ar 20 mL/min H_2_O-Distilled Water				
Laser Power [W]	Gas Products			
	H_2_ [%]	O_2_ [%]	CO_2_ [%]	CO [%]
10.0	47.00	10.44	11.23	31.33
9.0	54.42	6.80	11.56	27.21
8.0	54.30	9.05	9.50	27.15
7.0	53.25	11.83	11.24	23.67
6.0	55.56	7.94	12.70	23.81

**Table 2:** The ratio of gas products of laser-induced decomposition of 1% saline in a function of applied laser power in an argon atmosphere.

Ar 20 mL/min H_2_O + 1% NaCl				
Laser Power [W]	Gas Products			
	H_2_ [%]	O_2_ [%]	CO_2_ [%]	CO [%]
10.0	79.95	8.13	3.66	8.27
9.0	78.99	8.52	4.27	8.21
8.0	78.48	8.43	4.85	8.23
7.0	78.21	8.93	5.10	7.75
6.0	77.81	9.07	5.42	7.70
5.5	80.91	8.17	4.84	6.08
5.0	59.21	14.47	6.58	19.74
4.5	57.14	22.86	8.57	11.43

## Data Availability

Not applicable.

## Sample Availability

Samples of the compounds are available from the authors.
